# Leptospirosis in sugarcane plantation and fishing communities in Kagera northwestern Tanzania

**DOI:** 10.1371/journal.pntd.0007225

**Published:** 2019-05-31

**Authors:** Georgies F. Mgode, Maulid M. Japhary, Ginethon G. Mhamphi, Ireen Kiwelu, Ivan Athaide, Robert S. Machang’u

**Affiliations:** 1 Pest Management Centre, Sokoine University of Agriculture, Morogoro, Tanzania; 2 Chato District Hospital, Geita, Tanzania; 3 Kilimanjaro Christian Medical University College, Tumaini University, Moshi, Tanzania; 4 Kagera Sugar Company Ltd, Kagera Tanzania; Baylor College of Medicine, UNITED STATES

## Abstract

**Background:**

Leptospirosis is a bacterial zoonotic disease of worldwide importance, though relatively neglected in many African countries including sub Saharan Africa that is among areas with high burden of this disease. The disease is often mistaken for other febrile illnesses such as dengue, malaria, rickettsioses and enteric fever. Leptospirosis is an occupational disease likely to affect people working in environments prone to infestation with rodents which are the primary reservoir hosts of this disease. Some of the populations at risk include: sugarcane plantation workers, wetland farmers, fishermen and abattoir workers. In this study we investigated the prevalence of antibodies against *Leptospira* among sugarcane plantation and factory workers, fishing communities as well as among rodents and shrews in domestic and peridomestic environments within the study areas.

**Methods:**

The study was conducted in Kagera region, northwestern Tanzania and it involved sugarcane plantation workers (cutters and weeders), sugar factory workers and the fishing community at Kagera Sugar Company in Missenyi district and Musira island in Lake Victoria, Kagera, respectively. Blood was collected from consenting human adults, and from rodents and shrews (insectivores) captured live using Sherman traps. Serological detection of leptospiral antibodies in blood serum was carried out by the microscopic agglutination test (MAT).

**Results:**

A total of 455 participants were recruited from the sugarcane plantation (n = 401) and fishing community (n = 54) while 31 rodents and shrews were captured. The overall prevalence of antibodies against *Leptospira* in human was 15.8%. Sugarcane cutters had higher seroprevalence than other sugar factory workers. Prevalent antibodies against *Leptospira* serovars in humans were against serovars Lora (6.8%), Sokoine (5.3%), Pomona (2.4%), Hebdomadis (1.1%) and Kenya (0.2%). Detected leptospiral serovars in reservoir hosts were Sokoine (12.5%) and Grippotyphosa (4.2%). Serovar Sokoine was detected both in humans and small mammals.

**Conclusion:**

Leptospirosis is a public health threat affecting populations at risk, such as sugarcane plantation workers and fishing communities. Public awareness targeting risk occupational groups is much needed for mitigation of leptospirosis in the study areas and other vulnerable populations in Tanzania and elsewhere.

## Author summary

Leptospirosis is caused by a spirochete bacterium of the genus *Leptospira*. The disease is worldwide distributed, although highly neglected in some parts of the developing world. This study investigated the prevalence of antibodies against *Leptospira* in sugarcane plantation workers, a fishing community and rodents and shrews in the Kagera region, northwestern Tanzania. Seventy two of the 455 (15.8%) screened participants were seropositive to leptospirosis. The seroprevalence was higher among sugarcane cutters (18.4%) than other plantation workers, and 15.4% of hospitalized patients in the plantation hospital were seropositive. Prevalence of antibodies against *Leptospira* in the fishing community was 14.8%. Antibodies against *Leptospira* serovars detected in humans with their respective proportions, in brackets, were Lora (6.8%), Sokoine (5.3%), Pomona (2.4%), Hebdomadis (1.1%) and Kenya (0.2%). In the small mammals, the most detected antibodies were against *Leptospira* serovar Sokoine (12.5%) and serovar Grippotyphosa (4.2%). These results show that leptospirosis is public health risk requiring attention of the health system as well as the agricultural sector for its management.

## Introduction

Leptospirosis is a public health concern especially in the tropical and subtropical countries where the environment is optimal for survival of pathogenic leptospires [1]. The annual morbidity and mortality caused by leptospirosis worldwide is estimated to be 14.7 cases per 100,000 population [2]. Globally, Oceania region has the highest disease burden (150.6 cases/100,000 population), South east Asia (55.5), Caribbean (50.6) and East Sub Saharan Africa (25.6) [2, 3]. In Tanzania the annual incidence is 75–102 cases per 100,000 population [4]. Rodents are considered major reservoirs of *Leptospira* [5] and other wild animals and birds found in wetland areas may also carry and spread leptospires into the surroundings [6]. The disease is associated with certain occupational activities such as rice and sugarcane farming, fishing and fish farming, livestock keeping, handling animal products and water sports [7, 8]. Males are most affected than females contributing to 80% of the total burden [3]. Humans can be infected through contact with urine or other materials from infected animals or contaminated water and soil [9]. In Tanzania, leptospirosis has been reported in patients with non-malaria fevers [10, 11] and in animals including rodents and domestic animals [12–15]. Antibodies against *Leptospira* have been demonstrated also in freshwater fishes [6] in Tanzania suggesting potential risk to fishermen and people undertaking irrigation activities such as, rice farming and sugarcane plantation. Studies on leptospirosis in these at risk populations are lacking, hence the burden of leptospirosis in fishing communities and sugarcane plantations is not known. Sugarcane plantation and rice farming are important agricultural sectors in Tanzania, which engage permanent and seasonal workers from different parts of the country. Understanding the burden of leptospirosis in these occupational groups could provide baseline information needed for informing policy, especially because the disease is neglected and rarely considered for diagnosis in the health system [16]. In this study we investigated the serological prevalence of leptospirosis in selected risk populations of sugarcane plantation workers and fishing in northwestern Tanzania Also, we identified potential *Leptospira* serovars circulating in the region, which would serve as important antigens for diagnostic purposes.

## Methods

### Study location

**Fig 1:**
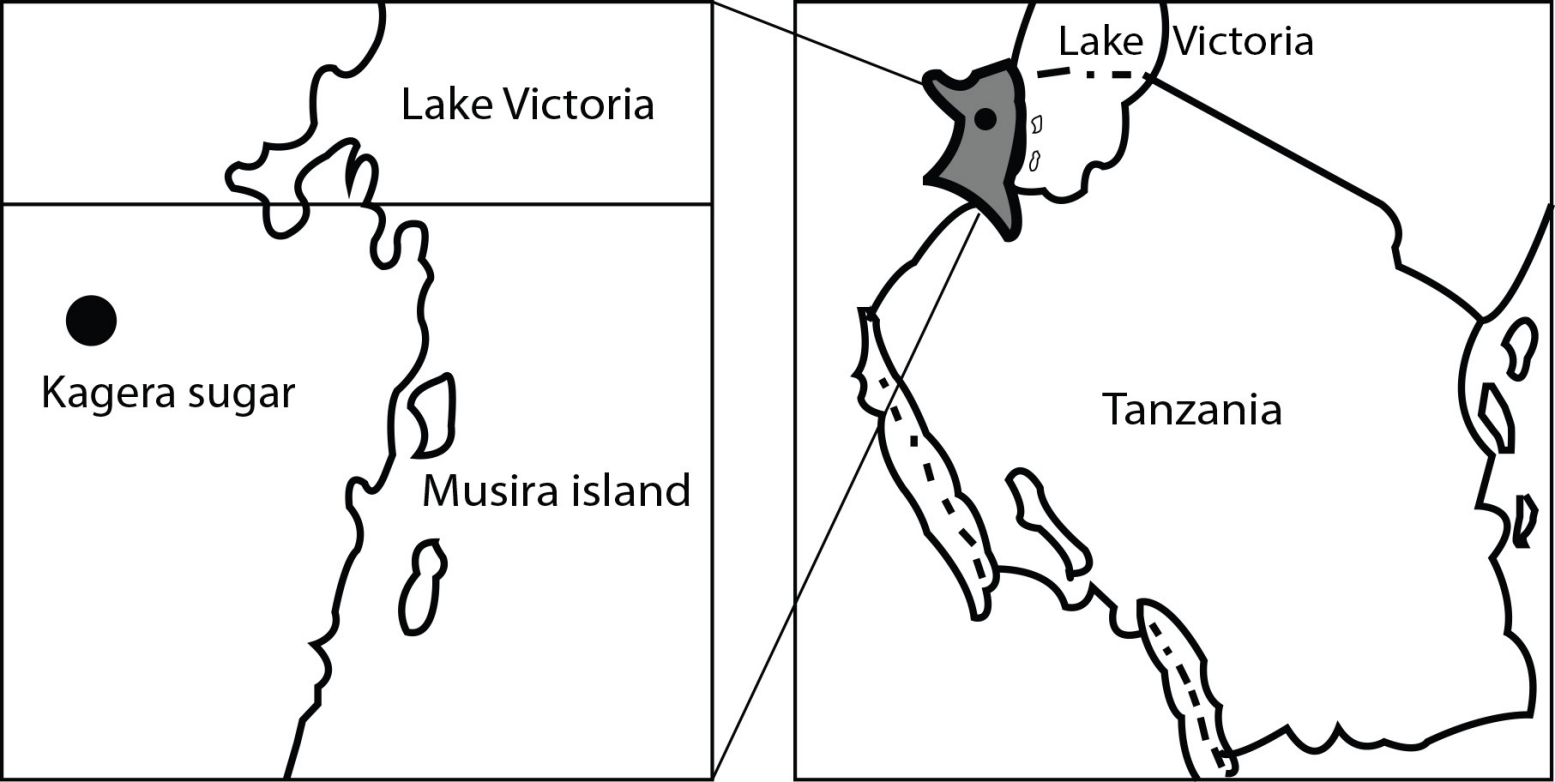
Map of Tanzania showing location of Kagera region and the study locations of fishing community (Musira island) and sugarcane plantation at Kagera sugar company.

This study was conducted in Kagera region northwestern Tanzania at Musira island (S 01° 19.914’, E 031° 49.772’ with elevation of 1120 meter above sea level, and at Kagera sugar company (S 01° 12.807, E 031° 16.510’) with elevation of 1157 meter above sea level. Kagera region receives bi-modal rainfall pattern ranging between 900–2,000 mm per annum, temperatures range between 20°C and 28°C. Kagera region is located along Lake Victoria hence fishing is among major socio-economic activity apart from large scale sugarcane plantation. Kagera Sugar Company is one of the biggest sugarcane plantations in the country. The two study sites (fishing community and Musira and sugarcane plantation community at Kagera sugar company are 76.2 km apart (Fig 1).

### Study populations and sample size

#### Human subjects

Human leptospirosis is estimated to be 10% in Tanzania [10, 13, 15]. Sample size estimation formula used was N = Z^2^XP (1-P)/δ^2^, whereby; N = Estimated sample size, Z = Standard normal variate (1.96) for 95% confidence level, P = Proportion (prevalence), δ = Precision or absolute error (~ 0.05) for 95% confidence level. N = 1.96^2^x0.1 (1–0.1)/0.05^2^ = 138 + 15% of 138 = 160

To increase the power of the study to 80%, 455 of the human study population were sampled from sugarcane plantation workers/cutters and fishermen along Lake Victoria in Kagera region. Sugarcane cutters make the largest proportion of the Kagera sugar company staff. Other employees include those engaged with management, sugarcane planting, weeding, fertilizer application, herbicides application, health services and security. There were 924 sugarcane cutters (seasonal employees) eligible for the study whereas 401 (43.4%) agreed to participate in the study following sensitization about leptospirosis disease. Encampment residents including petty traders in the sugarcane plantation community as well as hospitalized patients were also studied.

Other study participants were from the fishing community at Musira island located near Bukoba town, which were predominantly fishermen.

#### Inclusion and exclusion criteria for human subjects

The study included adult sugarcane plantation workers/cutters, weeders, and factory workers working in the sugarcane plantation or encampment residents residing in Kagera sugar company compounds; fishermen and other willing individuals living in the Musira island fishing community. Individuals under 18 year old and those who did not agree to sign informed consent were excluded from the study. Participants were informed about the study at gatherings held in residential camps of Kagera Sugar Company while in the fishing community, individuals were informed and at a public gathering called by the village authority/ chairman. Information about leptospirosis disease and how it is transmitted to humans was communicated to villagers at this gathering to sensitize and increase the participation rate in the study. No interviews were conducted with participants to ascertain whether recalled history of clinical illness related with leptospirosis since majority were assumed likely to agree to this because fevers are common in this tropical region.

#### Hospitalized participants

There were 58 patients hospitalized at Kagera sugar hospital during the sampling period. These were informed about leptospirosis whereby 13 (22.4%) patients with fevers agreed to provide blood for testing antibodies against leptospirosis.

Blood samples (2–4 ml) were aseptically collected by medical personnel using sterile 5 ml vacutainer tubes and kept in a refrigerator overnight to separate serum. Un-separated blood was centrifuged at 3000g for 10 min to obtain the serum for microscopic agglutination test (MAT).

#### Rodents and shrews

Animal sample size was estimated by using 8% which is the isolation success rate of *Leptospira* from rodents and insectivores reservoir hosts previously reported from Tanzania [15] using same formula used for estimating human subjects sample size, i.e. N = Z^2^XP (1-P)/δ^2^ where by N = estimated sample size, Z = standard normal variate (1.96) for 95% confidence level, P = isolation success rate (prevalence), δ = Precision or absolute error (~ 0.05) for 95% confidence level (N = 1.96^2^X0.08 (1–0.08)/0.05^2^ = 113+ 15% of 113 = 130. Therefore, 130 rodents and insectivores were required for this study.

Rodents and shrews (*Crocidura* spp.) were captured live using Sherman live traps. The rodents and shrews were then anaesthetized using di-ethyl ether. Blood (1 to 2 ml) was aseptically collected from the supraorbital vein and/ or through heart puncture using capillary tubes and sterile syringes and needles, respectively. The blood was immediately transferred into plain 2.5 ml Eppendorf tubes and allowed to clot to obtain serum samples which were then shipped in cool boxes to the Leptospirosis Research Laboratory at Pest Management Centre, Sokoine University of Agriculture where was kept frozen at -20°C until used.

#### Serological detection of leptospiral antibodies

Antibodies against *Leptospira* in humans and animals were detected using MAT as previously described [17, 18]. Live leptospiral antigens consisted of six serovars were used, four of which are local isolates, namely *L*. *kirschneri* serovar Sokoine, *L*. *borgpetersenii* serovar Kenya, *L*. *interrogans* serovar Lora and *L*. *kirschneri* serovar Grippotyphosa [1, 14, 15, 19]. The remaining two (*L*. *interrogans* serovars Hebdomadis and *L*. *interrogans* serovar Pomona) were reference isolates initially supplied by the Royal Tropical Institute (KIT Biomedical Research, WHO/FAO/OIE Leptospirosis Reference Centre), Amsterdam, the Netherlands. These leptospires were grown in Ellinghausen and McCullough–Johnson and Harris, (EMJH) culture medium for 5 to 8 days while monitoring for growth density and purity using dark field microscope. Live leptospiral antigens with density 3×10^8^ leptospires/ml were used in MAT as recommended elsewhere [17, 18]. Briefly, 10μl of serum was mixed with 90μl of phosphate buffered saline (PBS) (pH 7) and then serially double diluted with PBS diluted further to obtain initial dilutions of 1:10, 1:20, 1:40 and 1:80. Volume of 50μl of well grown live leptospires antigen were then added to each well to obtain final dilutions of 1:20, 1:40, 1:80 and 1:160 recommended for initial screening. Positive samples at ≥ 1:20 were diluted further and retested to determine cut-off agglutination titres, which were those in which 50% of the leptospires agglutinated compared with the negative control in which only PBS and the live cultures were mixed [17, 18]. The highest dilution in screening was 1:160 whereas in titration was 1:20480.

#### Isolation of *Leptospira* from rodents and insectivores

The isolation of *Leptospira* from rodents and insectivores/shrews was done by aseptically culturing the kidney homogenate and urine into Fletcher culture media as previously described [13, 15]. Briefly, two drops of fresh urine were inoculated into a screw capped tube containing sterile semisolid *Leptospira* Fletcher’s culture medium prepared according to manufacturer’s instructions and containing 50mg/ml of 5-Flourouracil selective inhibitor. Kidney specimens were placed into a sterile vials containing sterile phosphate buffered saline (pH 7.0) and homogenized using sterile syringe needle cap as previously described [13, 15]. Two drops of kidney homogenate were aseptically inoculated into Fletcher medium and incubated at room temperature while in field and at 30°C in the laboratory for 8 up to 20 weeks. Cultures were observed for leptospiral growth under dark field microscope every 7 days.

#### Data analysis

Prevalence of antibodies against *Leptospira* serovars tested were used to compare various at-risk groups and whether gender, occupation and age were risk factors. Various age groups were compared as well as occupation groups. Statistical significance of differences between proportions of prevalence of antibodies against *Leptospira* was determined using chi-test and associations were considered statistically significant when P-values were ≤ 0.05. Statistical analyses were conducted using MedCalc [20–22]. MedCalc uses the "N-1" Chi-squared test according to Campbell [20] and Richardson [21], whereas confidence interval is calculated according to Altman and co-workers [22].

### Ethical consideration

The ethical clearance for conducting this study was obtained from the Medical Research Coordinating Committee of the National Institute for Medical Research (NIMR), Certificate No. NIMR/HQ/R.8a/Vol.IX/2453, as well as from the Kilimanjaro Christian Medical University College, Research Ethics and Review Committee (CRERC), Moshi Tanzania (Ref. No. 993). Permission was also sought from local authorities in the study area.

## Results

### Humans–sugarcane plantation workers and fishing community

A total of 455 participants were sampled of which 401 (132 females and 269 males) were from sugarcane plantation and 54 (16 females and 38 males were from the Musira fishing island in Lake Victoria. The demographic profile of human participants was as shown in Table 1.

**Table 1:** Demographic information of human participants (n = 455).

	Participants number and proportion (%) to sample size	*Leptospira* positive and percentage	Comparison of differences in seroprevalence between categories	P-value
Human participants	455	72 (15.8)		
Gender				
Male	307 (67.5)	55 (17.9)	Male and females (6.4%) 95% CI = -0.8658 to 12.6811	0.0800
Female	148 (32.5)	17 (11.5)		
Age distribution				
18–37	366 (80.4)	53 (14.5)	18–37 year old and 38–57 year old group (2.2%) 95% CI = -5.5629 to 12.5150	0.6206
38–57	78 (17.1)	13 (16.7)	18–37 year old and ≥58 year old (40.0%) 95% CI = 13.1776 to 64.4124	0.0003
≥58	11 (2.4)	6 (54.5)	38–57 year old and ≥58 year old (37.8%) 95% CI = 9.5238 to 62.8984	0.0044
Occupation/category				
Fishing	54 (11.9)	8 (14.8)	Fishing and sugarcane cutters (3.6%) 95% CI = -8.8082 to 12.0938	0.5241
			Fishing and others-unexposed group (9.3%) 95% CI = -1.2424 to 21.5463	0.0777
Sugarcane cutters	315 (69.2)	58 (18.4)	Sugarcane cutters and others-unexposed group (12.9%) 95% CI = 4.1963 to 18.6242	0.0068
Hospitalized patients	13 (2.9)	2 (15.4)	Hospitalized and others–unexposed group (9.9%) 95% CI = -3.6319 to 36.9580	0.1999
Others	73	4 (5.5)		
Hospital staff	7 (1.5)	1 (14.3)	Hospitalized and hospital staff (1.1%) 95% CI = -37.5454 to 30.4008	0.9490
Security guards	20 (4.4)	0 (0.0)		
Office cleaners	15 (3.3)	1 (6.7)		
Shopkeepers	16 (3.5)	1 (6.3)		
Encampment Residents	15 (3.3)	1 (6.7)		

### Rodents and insectivores

The majority of rodents were *Rattus* spp. (55%) trapped indoors. Other rodents trapped included forest species (*Lophuromys* spp.) captured in the bushes near the sugarcane plantation and *Arvicanthis* spp. found in fallow land near sugarcane fields (Table 2).

**Table 2:** Rodents and insectivores collected in Kagera study sites.

Animal species	Number captured	Habitat	Proportion	Tested	Positive (%)	P-value
*Arvicanthis* spp.	1	Fallow/forest	3.2	1	0 (0.0)	
*Dasmys* spp.	1	Forest	3.2	1	0 (0.0)	
*Lophuromys* spp.	3	Forest	9.7	3	0 (0.0)	
*Rattus rattus*	17	House	54.8	16	3 (18.7)	0.5792^#^
*Crocidura* spp.	8	Forest	25.8	3*	1 (33.3)	
*Mus* spp.	1	Forest	3.2	0	n/a	

^*^Serum was not available for MAT from 5 shrews (insectivores) and Muss spp. which died in the trap before blood collection

^#^ Comparison of seroprevalence in *Rattus* spp. and insectivore (*Crocidura* spp.)

### Leptospirosis seroprevalence in humans

Prevalence of human leptospirosis in the two study populations of sugarcane plantation workers and fishing community was 15.8%. Fifty eight of the 317 (18.3%) sugarcane cutters were seropositive compared to 8 out of 54 (14.8%) of the fishing community subjects. Two of the 13 (15.4%) hospitalized patients were seropositive while other participants including office cleaners, petty traders and security guards contributed to 7.0% seropositivity (Table 1).

**Fig 2:**
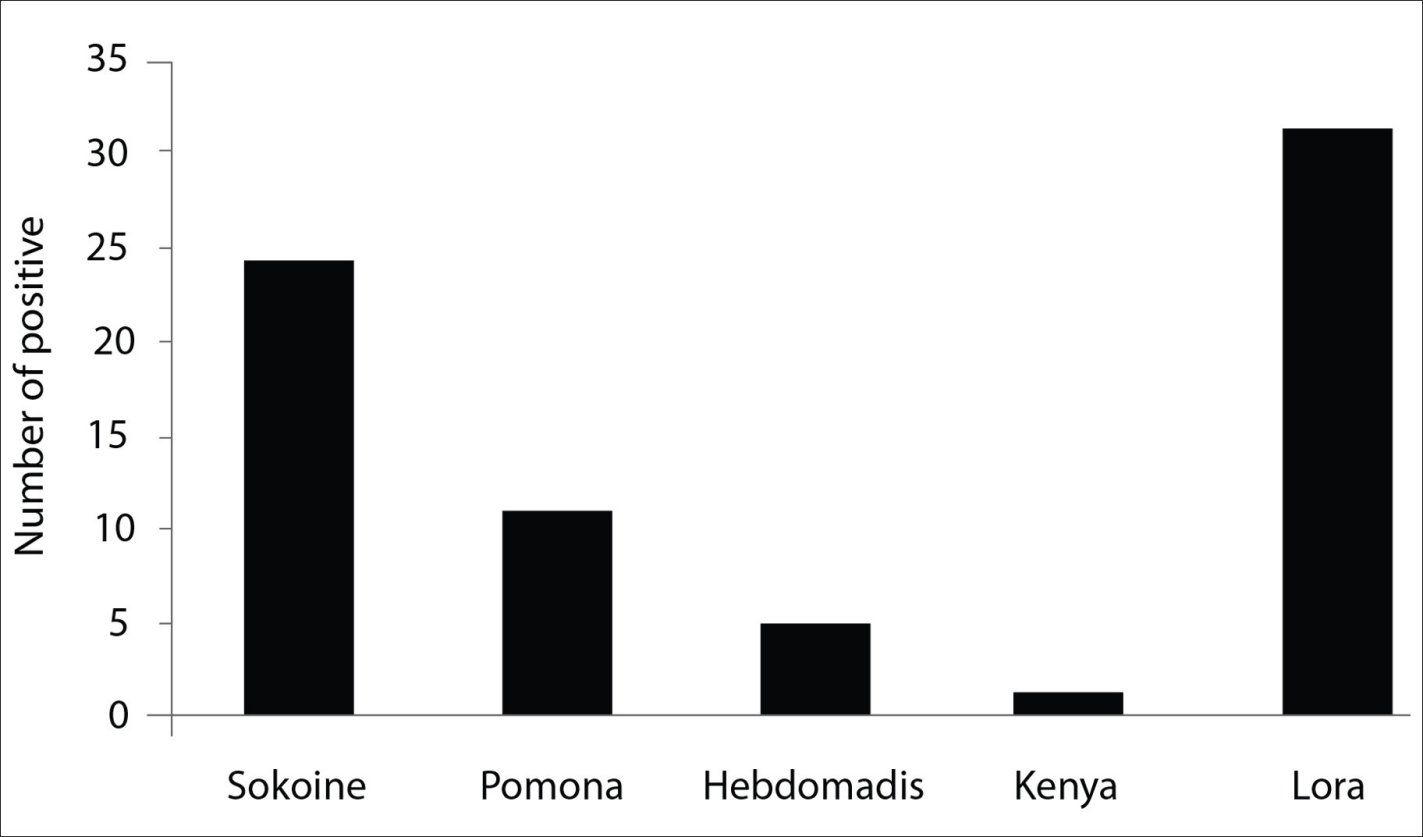
Antibodies against *Leptospira* serovars detected in humans.

Antibodies against tested *Leptospira* were relatively lower in rodents than in humans. The highest titre (1:2560) was observed in two individuals against serovar Pomona (Fig 2).

### Comparison of antibody prevalence in different groups

Prevalence of antibodies against *Leptospira* among different occupational groups, populations, gender and age groups were compared to determine whether certain groups were at more risk than others. The prevalence of antibodies against *Leptospira* between male and female participants was 6.4%, which was not statistically significant (p = 0.0800, 95% CI = -0.8658 to 12.6811, χ2 = 3.065, df = 1). The prevalence of antibodies against *Leptospira* in participants in age group of 18–37 year and 38–57 year old differed by 2.2% which was not statistically significant (p = 0.6206, 95% CI = -5.5629 to 12.5150, χ2 = 0.245, df = 1). There was significant difference in percentage of positive individuals (40%) between participants in age group of 18–37 year and ≥58 year old (40.0%) (p = 0.0003, 95% CI = 13.1776 to 64.4124, χ2 = 12.898, df = 1). Age group of 38–57 yrs and >58 year old also showed significant difference in percentage of positives (37.8%) that was also statistically significant (p = 0.0044, 95% CI = 9.5238 to 62.8984, χ2 = 8.105, df = 1). The prevalence of antileptospiral antibodies between fishing community and sugarcane cutters was 3.6% that is not statistically significant (p = 0.5241, 95% CI = -8.8082 to 12.0938, χ2 = 0.406, df = 1). Comparison of positive rate found in fishing community and unexposed group (others) showed 9.3%, which was not statistically significant (p = 0.0777, 95% CI = -1.2424 to 21.5463, χ2 = 3.111, df = 1). The difference in positive rate between sugarcane cutters and unexposed group (others) was 12.9%, which was statistically significant (p = 0.0068, 95% CI = 4.1963 to 18.6242, χ2 = 7.329, df = 1). Hospitalized participants and unexposed group (others) showed a difference of 9.9% that was not statistically significant (p = 0.1999, 95% CI = -3.6319 to 36.9580, χ2 = 1.643, df = 1). Hospitalized participants and hospital staff also showed a difference of 1.1% that was not significant (p = 0.9490, 95% CI = -37.5454 to 30.4008, χ2 = 0.004, df = 1).

#### Anti-leptospiral antibodies prevalence in rodents and shrews

Twenty-four rodents and shrews (*Crocidura* spp.) of the 31 animals collected around sugarcane plantations, forest and houses at Musira island and Kagera sugar company were tested for leptospiral antibodies. Four of the 24 animals (16.7%) were seropositive. Antibodies against *Leptospira* serovar Gripptyphosa was detected in one *Rattus rattus* rodent species (4.2%) whereas antibodies against *L*. serovar Sokoine was detected in two *Rattus rattus* and one insectivore (*Crocidura* spp.) (13%). Comparison of positive rates found in rodents and insectivores was not statistically significant (difference = 14.6%, 95% CI = -21.8152 to 62.0923, X^2^ = 0.307, df = 1, p = 0.5792). Antibodies against *L*. serovar Kenya, Lora, Pomona and Hebdomadis were not detected in rodents nor insectivores.

The seropositivity shows that antibodies against certain *Leptospira* serovars were detected either in humans or animals, while some were found in both humans and animals (Table 3). *Leptospira* serovar Sokoine had higher seropositivity rate in both humans (5.3%) and animals (12.5%) unlike the other serovars (Pomona, Grippotyphosa, Hebdomadis and Lora) detected in humans or animals only (Table 3). The overall prevalence of leptospirosis in rodents and insectivores was 16.7%, of which rodents contributed 12.5% (3/24) and insectivores 4.2% (1/24) (Table 3) The overall difference in prevalence of antibodies against Leptospira between humans and rodents was 0.9% (95% CI = -9.7420 to 20.3338, χ2 = 0.014, df = 1, p = 0.9064) whereas the prevalence of antibodies against *Leptospira* serovar Sokoine which occurred in both humans and rodents differed between the two groups by 7.2% (95% CI = -1.3180 to 25.7827, χ2 = 2.208, df = 1, p = 0.1373). The differences in seroprevalence of leptospiral antibodies in humans and rodents as well as of serovar Sokoine were not statistically significant (Table 3).

**Table 3:** *Leptospira* serovars with antibodies detected in humans (n = 455), rodents and shrews (n = 24).

*Leptospira* serovars	Positive and percentage		Statistical value
	Humans	Rodents and shrews	
*L*. *interrogans* serovar Pomona	11 (2.4)	0	
*L*. *kirschneri* serovar Grippotyphosa	0	1 (4.2)	
*L*. *borgpetersenii* serovar Kenya	1 (0.2)	0	
*L*. *kirschneri* serovar Sokoine	24 (5.3)	3 (12.5)*	0.1373
*L*. *interrogans* serovar Lora	31 (6.8)	0	
*L*. *interrogans* serovars Hebdomadis	5 (1.1)	0	
Overall positives	72 (15.8)	4 (16.7)	0.9064

*Three positive animals against *Leptospira* serovar Sokoine included one insectivore and two rodents.

*Leptospira* isolation from rodents and insectivores’ urine and kidney homogenates did not yield positive cultures.

## Discussion

This study shows high prevalence of antibodies against *Leptospira* in humans involved in sugar production and fishing in the Kagera region, northwestern Tanzania. Leptospirosis in rodents and shrews captured in the areas is also reported.

Findings suggests that sugarcane plantation workers especially sugarcane cutters and fishing communities are potentially at risk. A prevalence of 15.8% was found in sugarcane plantation workers, with cane cutters having the higher prevalence of 18.4%, followed by other plantation workers and hospitalized patients. Prevalence of anti-leptospiral antibodies was also high (14.8%) in fishermen and other individuals living on the Musira island, which is a fishing island. This suggests that fishing communities can get leptospirosis following contact with water contaminated with urine of the reservoir hosts. The prevalence of human leptospirosis in sugarcane plantation workers reported in this study (18.4%) is lower than that reported in sugarcane plantation workers in central America (59%) [19] but higher than the 0.7% prevalence reported from Trinidad and Tobago [23].

Prevalence of antibodies against *Leptospira* among different occupational groups, populations, gender and age groups showed variations suggesting that individuals belonging to certain groups and occupation groups have different levels of risk of contracting leptospirosis. For example, while there was no significant difference in the prevalence of leptospiral antibodies between male and females despite that the study had more males than females due to the nature of the occupation of the study populations, there was a significant difference in prevalence of antibodies against *Leptospira* found in participants in two age groups of 18–37; 38–57 year old versus participants with age above 58 year old. Findings show that participants with over 58 year old have significantly higher proportion of antibodies against *Leptospira* than those with age below 58 year old (i.e. 18–37; 38–57 year old). This could be probably associated with potential prolonged exposure to risk environment such as sugarcane cutting for many years than newer entrants. The fishing community and sugarcane plantations appear to have similar risk levels since the prevalence of antibodies in these two populations was not statistically significant. However, comparison of fishing community and sugarcane cutters considered risk populations with unexposed groups consisting of participants engaged with less risk activities such as office work, security and petty traders show that fishing community does not differ to the unexposed group while sugarcane cutters show more risk than unexposed group. This can be explained with fact that fishing community included the general population of the fishing island including school pupils and other residents likely to have various levels of risk of contracting leptospirosis while sugarcane cutters consisted a uniform group of individuals engaged with same activity of cutting sugar hence likely to have same level of risk higher than the general population.

The prevalent antibodies against *Leptospira* serovars found in humans were against *Leptospira interrogans* serovar Lora (6.8%), *L*. *kirschneri* serovar Sokoine (5.3%) and slightly *Leptospira interrogans* serovar Pomona (2.4%). *Leptospira interrogans* serovar Hebdomadis and *L*. *borgpetersenii* serovar Kenya were least found with prevalence of 1.1% and 0.2%, respectively. *Leptospira kirschneri* serovar Sokoine and *L*. *kirschneri* serovar Grippotyphosa were frequently found in both humans and animals as previously reported [8, 15] in agro-pastoralists communities living in Katavi-Rukwa ecosystem [8] indicating a wider distribution of leptospirosis in Tanzania.

These findings shows that the roof rat (*Rattus* spp.) is an important reservoir of leptospirosis in Kagera region as demonstrated by high positivity rate among the house rats collected in different localities in the study areas. Comparison of positive rates found in the roof rats and an insectivore showed no statistically significant difference due to small sample size of rodents and shrews collected. A larger sample size estimated for this study was not achieved due to seasonal variations in rodent populations hence suggesting further sampling to enhance robust determination of the major reservoir of *Leptospira* in this region. Antibodies against *L*. serovar Kenya, Lora, Pomona and Hebdomadis were not detected in rodents nor insectivores. The rats were seropositive against *L*. *kirschneri* serovars Sokoine and *L*. *kirschneri* serovar Grippotyphosa. Rodents had lower antibody titres (1:20–1:40) than humans in which higher titres up to 1:2560 were determined by MAT which is the gold standard test for leptospirosis diagnosis [9, 24]. High antibody titres against *Leptospira* serovars detected in humans suggest the existence of recent infections.

The predominant circulating *Leptospira* serovars which antibodies against was detected in humans, namely *Leptospira interrogans* serovar Lora, *L*. *kirschneri* serovar Sokoine, *L*. *interrogans* serovar Pomona, *L*. *interrogans* serovar Hebdomadis and *L*. *borgpetersenii* serovar Kenya have been previously reported in humans, rodents and domestic animals [10, 15, 25]. *Leptospira kirschneri* serovar Sokoine was mainly found in both humans and animals in Tanzania whereas *L*. *interrogans* serovar Grippotyphosa was mainly detected in the reservoir hosts. *Leptospira interrogans* serovar Lora was not detected in rodents, indicating potential diversity of sources of human infection. It is known that certain *Leptospira* serovars demonstrate host-specificity and might be absent in certain rodent species [15]. Further investigations are needed to establish the source or reservoir hosts of serovar Lora in the study areas and to determine whether the plantation workers who also come from outside Kagera had leptospirosis exposure prior to their recruitment at the sugarcane plantation. This could be achieved by including leptospirosis screening during general health examinations performed before recruiting cane cutters.

The observed high prevalence of leptospirosis in the fishing community corroborate previous report of high seropositivity/leptospiral antibodies in freshwater fishes and thus potential risk of leptospirosis in fishing communities and in people working in the fishing industry [15, 19, 23]. It is recommended that leptospirosis control should include rodent management, and public awareness. Furthermore, leptospirosis screening should be introduced in risk occupational groups in Tanzania and elsewhere where the disease is neglected [16]. Detection of leptospiral antibodies in hospitalized patients during this study indicates further the importance of considering leptospirosis among febrile illnesses that are non-malarial. The prevalence of 15.4% of leptospirosis in hospitalized patients corroborates previous reports from northern Tanzania and Morogoro among hospitalized patients with febrile illness [10, 11, 26]. This further emphasizes the need to include leptospirosis in differential diagnosis of febrile illnesses.

Further surveillance studies are needed to isolate and characterize the disease causative *Leptospira* serovars beyond serological surveillance. These should include cross agglutination absorption test, and molecular typing [25]. The major limitations of this study were failure to isolate the causal agent, which would have enabled its characterization. Similarly, future studies should include larger populations of potential reservoirs.

Leptospirosis is a public health threat in sugarcane plantation workers and the fishing communities. Preventive measures are needed to mitigate risks of leptospirosis. These should include rodent control, public awareness and screening for leptospirosis in individuals with non-malarial fevers [16] and vulnerable occupational groups such as sugarcane cutters. *Leptospira* serovars Lora, Sokoine, Pomona, Hebdomadis, Kenya and Grippotyphosa should be included as antigens for broader leptospirosis screening in humans and animals from this region.
